# Supplementary material for: Multidimensional impacts of coronavirus pandemic in adolescents in Pakistan: A cross sectional research
Source: PLoS One. 2022 Jan 5;17(1):e0262325. doi: 10.1371/journal.pone.0262325 (PMC8730410; doi:10.1371/journal.pone.0262325)
Supplement: S1 File — (PDF) [file pone.0262325.s001.pdf]

Multidimensional impacts of coronavirus pandemic in adolescents in Pakistan: A cross sectional research

SUPPORTING FILE

Table S1 : ***Epidemic Pandemic Impact Inventory-Adolescent Domains Total Mean Scores of Participants and subgroups (N=842)***

	**Gender**					**Age**			**Area of Residence**			
**EPII-A Subscales**	**Women (n=707)**	**Men (n=135)**			**<15 Years (n=108)**	**15-18 years (n=734)**			**Urban (n=677)**	**Rural (n=165)**		
	Mean (SD)	***Mean (SD)***	$t_{(841)}$	p	***Mean (SD)***	***Mean (SD)***	$t_{(???)}$	p	***Mean (SD)***	***Mean (SD)***	$t_{(841)}$	p
Work & Employment	3.62 (3.35)	5.16 (3.20)	4.90	0.470	3.23 (2.72)	3.96 (3.45)	-1.50	0.000	3.73 (3.28)	4.45 (3.69)	-0.68	0.006
Education & Training	4.64 (2.92)	6.24 (2.58)	5.95	0.030	4.50 (2.37)	4.95 (3.00)	-2.10	0.000	4.86 (2.90)	5.04 (3.05)	-2.49	0.060
Home life	4.31 (2.81)	4.86 (2.27)	2.13	0.00	4.06 (2.13)	4.45 (2.82)	-1.35	0.000	4.22 (2.65)	5.13 (2.98)	-3.87	0.027
Social Activities	4.84 (4.11)	6.90 (3.80)	5.39	0.030	4.21 (3.15)	5.31 (4.23)	-2.59	0.000	5.06 (4.02)	5.62 (4.51)	-1.55	0.009
Economics	1.74 (1.73)	1.76 (1.69)	.15	0.250	1.23 (1.56)	1.82 (1.74)	-3.31	0.005	1.68 (1.69)	1.99 (1.83)	-2.09	0.064
Emotional health & Well being	2.39 (2.48)	4.33 (2.95)	8.06	0.000	2.21 (2.11)	2.78 (2.73)	-2.05	0.000	2.69 (2.63)	2.78 (2.78)	-0.39	0.316
Physical health problems	2.65 (3.16)	5.23 (3.99)	8.31	0.000	2.45 (2.15)	3.15 (3.58)	-1.97	0.000	2.96 (3.37)	3.50 (3.66)	-1.83	0.047
Physical distance & Quarantine	1.67 (2.12)	2.87 (2.28)	5.92	0.040	1.44 (1.72)	1.93 (2.24)	-2.14	0.000	1.80 (2.14)	2.13 (2.38)	-1.76	0.053
Infection History	1.10 (2.55)	3.13 (3.45)	7.99	0.000	1.59 (2.62)	1.40 (2.84)	0.67	0.901	1.39 (2.71)	1.54 (3.20)	-0.59	0.190
Positive Impact	9.37 (6.35)	8.90 (4.87)	-.815	0.000	8.46 (4.22)	9.41 (6.37)	-1.50	0.001	9.18 (5.96)	9.76 (6.81)	-1.10	0.012

The mean (standard deviation) for all domains of EPII-A are presented in Table . Overall, male adolescents consistently had significant negative impact in most EPII-A domains in comparison to females (P-value<.05). Compared with adolescents aged upto 15 years, 15-18 years old respondents reported higher mean scores on most domains. Only a few domains were significantly affected by urban/ rural areas**.** Adolescents having past medical illness and past psychiatric illness had higher scores in infection history (P value<.001). Adolescents with history of psychiatric illness had higher mean scores in emotional health and well-being domains (4.60 ± 3.60 vs 2.94 ± 3.39) but it was not statistically significant.

Table S2: **Relationship between Age, Gender and Different Domains of EPII-A (N=842)**

S.N	Variables	2	3	4	5	6	7	8	9	10	11	12	M (SD)
1	**Age**	**.14****	**.01**	**.09****	**.06**	**.15****	**.10****	**.10****	**.09***	**.17****	**.01**	**.12****	**17.98 (2.96)**
2	**Gender**	-	**-.20****	**-.17****	**-.07***	**-.18****	**-.00**	**-.27****	**-.28****	**-.20****	**-.27****	**.04**	**1.89 (.36)**
3	**Work & Employment EPII-A**		-	.42**	.45**	.43**	.23**	.40**	.39**	.32**	.17**	.17**	**4.90 (2.93)**
4	**Education & Training EPII-A**		-	-	.54**	.51**	.37**	.49**	.45**	.42**	.29**	.15**	**3.87 (3.37)**
5	**Home Life EPII-A**		-	-	-	.51**	.50**	.45**	.41**	.35**	.22**	.23**	**4.40 (5.17)**
6	**Social Activities EPII-A**		-	-	-	-	.44**	.55**	.53**	.46**	.36**	.24**	**1.74 (1.72)**
7	**Economic EPII-A**		-	-	-	-	-	.39**	.36**	.25**	.24**	.23**	**2.70 (2.65)**
8	**Emotional Health & Well-being EPII-A**		-	-	-	-	-	-	.72**	.51**	.45**	.12**	**3.06 (3.43)**
9	**Physical Health Problems EPII-A**		-	-	-	-	-	-	-	.55**	.49**	.14**	**1.86 (2.18)**
10	**Physical distance EPII-A**		-	-	-	-	-	-	-	-	.44**	.22**	**1.42 (2.81)**
11	**Infection History EPII-A**		-	-	-	-	-	-	-	-	-	.09**	**7.17 (4.72)**
12	**Positive Change EPII-A**		-	-	-	-	-	-	-	-	-	-	

Note. $^{*}p<0.05$. $^{**}p<0.01$

Results revealed significant positive correlation between age, gender, education and training, social activities, economic conditions, emotional health and well-being, physical health problems, physical distance and positive change. There was significant negative correlation between gender (being female) and work and employment, education and training, home life, social activities, emotional health and well-being, physical health problems, physical distance and infection history. Different domains of EPII-A i.e., had significant correlation e.g.,

there was significant correlation between work and employment had significant correlation with education and training, home life, social activities, economics, emotional health and well-being, physical health problems, physical distance, infection history and positive change.
