# Supplementary material for: Multidimensional impacts of coronavirus pandemic in adolescents in Pakistan: A cross sectional research
Source: PLoS One. 2022 Jan 5;17(1):e0262325. doi: 10.1371/journal.pone.0262325 (PMC8730410; doi:10.1371/journal.pone.0262325)
Supplement: Attachment — Submitted filename: R2 Answer to Reviewer Query PONE.docx [file pone.0262325.s003.docx]

PONE-D-21-23130R1

Multidimensional impacts of coronavirus pandemic in adolescents in Pakistan: A cross sectional research

PLOS ONE

Journal Requirements:

Please review your reference list to ensure that it is complete and correct. If you have cited papers that have been retracted, please include the rationale for doing so in the manuscript text, or remove these references and replace them with relevant current references. Any changes to the reference list should be mentioned in the rebuttal letter that accompanies your revised manuscript. If you need to cite a retracted article, indicate the article’s retracted status in the References list and also include a citation and full reference for the retraction notice.

Additional Editor Comments:

Considering my own reading and reviewer suggestion, I am recommending minor revision before accepting this paper for publication in PLOS One.

[Note: HTML markup is below. Please do not edit.]

Reviewers' comments:

Reviewer's Responses to Questions

**Comments to the Author**

1. If the authors have adequately addressed your comments raised in a previous round of review and you feel that this manuscript is now acceptable for publication, you may indicate that here to bypass the “Comments to the Author” section, enter your conflict of interest statement in the “Confidential to Editor” section, and submit your "Accept" recommendation.

Reviewer #1: All comments have been addressed

2. Is the manuscript technically sound, and do the data support the conclusions?

The manuscript must describe a technically sound piece of scientific research with data that supports the conclusions. Experiments must have been conducted rigorously, with appropriate controls, replication, and sample sizes. The conclusions must be drawn appropriately based on the data presented.

Reviewer #1: Partly

3. Has the statistical analysis been performed appropriately and rigorously?

Reviewer #1: No

4. Have the authors made all data underlying the findings in their manuscript fully available?

The [PLOS Data policy](http://www.plosone.org/static/policies.action#sharing) requires authors to make all data underlying the findings described in their manuscript fully available without restriction, with rare exception (please refer to the Data Availability Statement in the manuscript PDF file). The data should be provided as part of the manuscript or its supporting information, or deposited to a public repository. For example, in addition to summary statistics, the data points behind means, medians and variance measures should be available. If there are restrictions on publicly sharing data—e.g. participant privacy or use of data from a third party—those must be specified.

Reviewer #1: Yes

5. Is the manuscript presented in an intelligible fashion and written in standard English?

PLOS ONE does not copyedit accepted manuscripts, so the language in submitted articles must be clear, correct, and unambiguous. Any typographical or grammatical errors should be corrected at revision, so please note any specific errors here.

Reviewer #1: Yes

6. Review Comments to the Author

Please use the space provided to explain your answers to the questions above. You may also include additional comments for the author, including concerns about dual publication, research ethics, or publication ethics. (Please upload your review as an attachment if it exceeds 20,000 characters)

Reviewer #1: How was the reliability and validity of the Pandemic-Adolescent Pandemic Impact Inventory determined? What were the hypotheses of the study and What were the assumptions of using the Mann Whitney U test and Logistic Regression?

EPII-A is a newly developed assessment measure and is still in the process of determining psychometric properties. (Morris, A.S., Ratliff, E. L., Grasso, D. J., Briggs-Gowan, M. J., Ford, J. D., & Carter, A.S. (2020). The Epidemic – Pandemic Impacts Inventory Adolescent Adaptation (EPII-A). University of Connecticut School of Medicine.)The authors of EPII-A has also invited researchers world widely to employ the inventory to find empirical evidences to determine psychometric properties of the inventory in general population. Main psychometric results of the EPII-Adolescents Adaptation version i.e., construct validity, EFA, item analysis and Cronbach’s alpha reliabilities in our population revealed good properties and are being reported separately.(unpublished Manuscript). Cronbachs alpha was 0.76 for most items of EPII Adolescent adaptation.

Study was exploratory in nature and main hypothesis we had in mind was that COVID-19 Pandemic has significant impact on adolescents in all domains of life, irrespective of gender, area of residence and other variables.

As data was skewed thus Mann whitney test is applied and median and interquartile ranges were reported. OLS was done as per one of the reviewers suggestions to create a link between the present literature and add on to the already published findings. Logistic regression analyses (enter method) were employed to predict demographic variables i.e., gender, residential area, family system and age groups as it allows to test models to predict binary categorical outcomes.

7. PLOS authors have the option to publish the peer review history of their article ([what does this mean?](https://journals.plos.org/plosone/s/editorial-and-peer-review-process#loc-peer-review-history)). If published, this will include your full peer review and any attached files.


If you choose “no”, your identity will remain anonymous but your review may still be made public.


**Do you want your identity to be public for this peer review?** For information about this choice, including consent withdrawal, please see our [Privacy Policy](https://www.plos.org/privacy-policy).

Reviewer #1: **Yes:** Mahmooda Aftab

[NOTE: If reviewer comments were submitted as an attachment file, they will be attached to this email and accessible via the submission site. Please log into your account, locate the manuscript record, and check for the action link "View Attachments". If this link does not appear, there are no attachment files.]

While revising your submission, please upload your figure files to the Preflight Analysis and Conversion Engine (PACE) digital diagnostic tool, <https://pacev2.apexcovantage.com/>. PACE helps ensure that figures meet PLOS requirements. To use PACE, you must first register as a user. Registration is free. Then, login and navigate to the UPLOAD tab, where you will find detailed instructions on how to use the tool. If you encounter any issues or have any questions when using PACE, please email PLOS at [figures@plos.org](mailto:figures@plos.org). Please note that Supporting Information files do not need this step.
